# Evaluation of the Clinical and Economic Effects of a Primary Care Anchored, Collaborative, Electronic Health Lifestyle Coaching Program in Denmark: Protocol for a Two-Year Randomized Controlled Trial

**DOI:** 10.2196/19172

**Published:** 2020-06-25

**Authors:** Carl J Brandt, Jeanette Reffstrup Christensen, Jørgen T Lauridsen, Jesper Bo Nielsen, Jens Søndergaard, Camilla Sortsø

**Affiliations:** 1 Research Unit for General Practice Department of Public Health University of Southern Denmark Odense Denmark; 2 Department of Business and Economics University of Southern Denmark Odense Denmark; 3 LIVA Healthcare Copenhagen Denmark

**Keywords:** type 2 diabetes management, digital behavioral coaching, lifestyle change, health behavior change, obesity, weight loss, interactive advice, participant engagement, quality of life

## Abstract

**Background:**

Obesity is linked to a number of chronic health conditions, such as type 2 diabetes, heart disease, and cancer, and weight loss interventions are often expensive. Recent systematic reviews concluded that app and web-based interventions can improve lifestyle behaviors and weight loss at a reasonable cost, but long-term sustainability needs to be demonstrated.

**Objective:**

This study protocol is for a 2-year randomized controlled trial that aims to evaluate the clinical and economic effects of a primary care, anchored, collaborative, electronic health (eHealth) lifestyle coaching program (long-term Lifestyle change InterVention and eHealth Application [LIVA] 2.0) in obese participants with and without type 2 diabetes. The program’s primary outcome is weight loss. Its secondary outcome is the hemoglobin A_1c_ (HbA_1c_) level, and its tertiary outcomes are retention rate, quality of life (QOL), and cost effectiveness. Analytically, the focus is on associations of participant characteristics with outcomes and sustainability.

**Methods:**

We conduct a multicenter trial with a 1-year intervention and 1-year retention. LIVA 2.0 is implemented in municipalities within administrative regions in Denmark, specifically eight municipalities located within the Region of Southern Denmark and two municipalities located within the Capital Region of Denmark. The participants are assessed at baseline and at 6-, 12-, and 24-month follow-ups. Individual data from the LIVA 2.0 platform are combined with clinical measurements, questionnaires, and participants’ usage of municipality and health care services. The participants have a BMI ≥30 but ≤45 kg/m2, and 50% of the participants have type 2 diabetes. The participants are randomized in an approximately 60:40 manner, and based on sample size calculations on weight loss and intention-to-treat statistics, 200 participants are randomized to an intervention group and 140 are randomized to a control group. The control group is offered the conventional preventive program of the municipality, and it is compared to the intervention group, which follows the LIVA 2.0 in addition to the conventional preventive program.

**Results:**

The first baseline assessments have been carried out in March 2018, and the 2-year follow-up will be carried out between March 2020 and April 2021. The hypothesis is that the trial results will demonstrate decreased body weight and that the number of patients who show normalization of their HbA_1c_ levels in the intervention group will be much higher than that in the control group. The participants in the intervention group are also expected to show a greater decrease in their use of glucose-lowering medication and a greater improvement in their QOL when compared with the control group. Operational costs are expected to be lower than standard care, and the intervention is expected to be cost-effective.

**Conclusions:**

This is the first time that an app and web-based eHealth lifestyle coaching program implemented in Danish municipalities will be clinically and economically evaluated. If the LIVA 2.0 eHealth lifestyle coaching program is proven to be effective, there is great potential for decreasing the rates of obesity, diabetes, and related chronic diseases.

**Trial Registration:**

ClinicalTrials.gov NCT03788915; https://clinicaltrials.gov/ct2/show/NCT03788915

**International Registered Report Identifier (IRRID):**

DERR1-10.2196/19172

## Introduction

### Background

With the incidence of chronic lifestyle-related diseases rapidly increasing, cost-effective management is needed [1]. Enabling individually tailored care at a low cost is the ambition when introducing lifestyle interventions as a cornerstone in the prevention and management of chronic diseases [2-6]. For example, type 2 diabetes is strongly related to weight gain in adult life, and pathophysiological studies have established how and why people with type 2 diabetes can be returned to normal glucose control and have shown that even marginal weight loss can have a large impact on disease progression, bringing between 46% and 54% of patients with type 2 diabetes in remission [5,7]. App and web-based interventions aiming to promote a healthy lifestyle have attracted much attention owing to their potential for scalability and accessibility, low cost, privacy and user control, usability in municipal settings, and opportunities for real-time modifications and interactive advice [3,8]. While systematic reviews have concluded that app and web-based interventions can improve lifestyle behaviors, the sustainability of these interventions has been shown to be variable, and long-term sustainability needs to be demonstrated [3,8-12]. The literature shows that the usage of personal feedback from a known health coach on users’ own registrations, group support, and different behavioral change techniques (BCTs) is superior when compared with more automated services, especially on combining BCTs with face-to-face meetings [13].

A collaborative electronic health (eHealth) tool has shown promising results in weight loss among patients with diabetes when implemented in real-life settings [14]. In this study, our aim is three-fold as follows: (1) to measurably demonstrate the effect of a primary care, anchored, collaborative, eHealth lifestyle coaching program (long-term Lifestyle change InterVention and eHealth Application [LIVA] 2.0) on weight loss and diabetes regulation (hemoglobin A_1c_ [HbA_1c_] level) in a strongly scientific and randomized controlled trial (RCT) setting; (2) to evidence the sustainability of a long-term intervention with follow-up measurements at 12 and 24 months; and (3) to assess the cost-effectiveness of a digital intervention in a municipal setting.

### Product Development

The collaborative eHealth tool (version 1.0) was a web-based solution that users accessed using an internet browser on their personal computers. The present version of the collaborative eHealth tool (version 2.0) called LIVA has been developed based on experiences from approximately 140,000 individuals, who used the collaborative eHealth tool 1.0, over a period of 15 years. Besides general experience from using the eHealth tool 1.0, the research team conducted several qualitative research studies with the following three important stakeholder groups within weight loss interventions: patients, general practitioners, and health professionals practicing in eHealth coaching (health coaches) [8,15-19]. The key findings from the qualitative interviews regarding the use of version 1.0 of the collaborative eHealth tool can be summarized as follows: (1) establishment of an empathic relationship with a health coach; (2) an intuitive design that enables ease of use for both users and health coaches; (3) different modes of communication channels allowing for active communication at all weight loss steps among users; and (4) an intuitive backend design, including a text and video library, and communication templates enabling the optimization of tailored personal quality coaching.

A user-driven approach to the design of both the user and coach interfaces has enabled ease of use and has eased communication flow [20], allowing for tailored communication between the health coaches and the end users at all steps of the weight loss program [21]. An important feature of the intervention continues to be the initial establishment of an empathic relationship between the user and the health coach, who delivers effective remote digital coaching based on the user’s own registration. Algorithms have not replaced health coaches, and instead, the features of the eHealth tool 1.0 enable individualized care at minimal effort from the health coach. Afshin et al concluded in 2016 that a direct interaction between a user and a health coach enhances the effectiveness of lifestyle interventions [3]. By establishing a personal relationship outside the digital environment, which is maintained through the platform with backend follow-ups, we believe that we are able to facilitate tailored care and sustained participant engagement over time with limited continued health coach input in the process of successfully changing and sustaining a lifestyle change [8,21].

### Effectiveness of the Collaborative eHealth Tools 1.0 and 2.0

With version 1.0 of the collaborative eHealth tool, we found that personal eHealth lifestyle coaching combined with various BCTs, such as tailored information, self-monitoring, lifestyle coaching, personal feedback, reminders, and face-to-face support, led to relevant weight loss during a 20-month period [21]. These results were confirmed in an English RCT in a municipality setting, showing weight loss of 5.4 kg among men with type 2 diabetes compared with weight loss of 2.8 kg in a control group receiving standard care [8]. A refinement of the collaborative eHealth tool 1.0 was implemented in 15 Danish municipalities between the summer of 2016 and the summer of 2017, with approximately 12,000 users on the eHealth platform. Besides smaller adjustments from the collaborative eHealth tool 1.0, version 2.0 is a smartphone solution, which is downloaded as an app called LIVA. A feasibility study among patients with type 2 diabetes using LIVA 2.0 in a cross-municipality setting demonstrated relevant weight loss of 4.7 kg among users who had been on the platform for over 90 days. Modeling the association between weight reduction and decreased health care costs indicated cost effectiveness in a municipal perspective 1 year after implementation [14].

### Objectives and Hypotheses

Based on our previous research, we hypothesize that eHealth lifestyle coaching with the use of LIVA 2.0 will be effective in improving diet and increasing physical activity levels, thus reducing weight and improving HbA_1c_ levels after 1 year of intervention, with a sustained effect over time. This will result in increased health and quality of life (QOL) and decreased societal costs [22-27]. We expect that municipalities will find the intervention to be a cost-effective alternative for secondary prevention targeted at citizens who are at risk of developing chronic diseases, such as severe obesity, type 2 diabetes, cardiovascular disease, and cancer, and for tertiary prevention among patients with chronic type 2 diabetes, reducing the progression of diabetes and even resulting in its complete remission. To be able to investigate effects and cost-effectiveness among obese participants and obese patients with type 2 diabetes from a municipal perspective, the two target groups of the intervention are as follows: (1) obese citizens at risk of developing chronic diseases, such as severe obesity, type 2 diabetes, cardiovascular diseases, and cancer and (2) obese patients with type 2 diabetes. The intervention is compared with the conventional preventive program that the municipality offers to these two target groups. Furthermore, we investigate associations between participant characteristics and the success of LIVA 2.0 in providing novel insights regarding the associations between participant characteristics and success with a digital lifestyle intervention from both a 1-year and over 1-year perspective.

### Research Questions Concerning Both Target Groups

With regard to the primary outcome, the research question is as follows: What is the effect of LIVA 2.0 on users’ weight? With regard to the secondary outcome, the research question is as follows: What is the effect of LIVA 2.0 on users’ HbA_1c_ levels? With regard to the tertiary outcomes, the research questions are as follows: (1) What is the effect of LIVA 2.0 on the need for medicine use? (2) What is the effect of LIVA 2.0 on users’ QOL? (3) What is the cost-effectiveness from a municipal perspective? (4) Which participant characteristics are decisive for the effect and sustainability of LIVA 2.0?

## Methods

### Study Design

A multicenter RCT with a 1-year intervention and 1-year retention period with collection of clinical and questionnaire data is supplemented with long-term register-based follow-up.

### Study Population, Inclusion Criteria, and Setting

Participants are recruited through advertising on social media platforms, general practices, and patient organizations. Potential participants need to meet the inclusion criteria of the study (Textbox 1). Participants who would like to participate and who think they meet the inclusion criteria can register at the app URL [28]. After registration, potential participants are contacted by phone and are sent an email with more written and detailed information about the study and with an invitation to a face-to-face baseline meeting with a LIVA health coach. The individual face-to-face baseline meeting is conducted over 1 hour with a trained health care professional, who is a clinical dietitian by profession and who has been working with eHealth lifestyle coaching for at least 2 years (referred to as the health coach). The face-to-face baseline meeting is scheduled to take place within 7 to 14 days after the information material is sent by email to the potential participant in order to ensure that the participant has sufficient time to reflect on the decision to participate in the study. At the face-to-face baseline meeting, the participant is invited to bring a friend or a family member. The meeting takes place at the research unit for general practice or in the municipality where the participant lives. At the meeting, the health coach confirms that the participant meets the inclusion criteria and explains the details of the study to the participant, and at the end of the meeting, the coach obtains signed informed consent if the potential participant still wishes to participate. Thereafter, the participant is measured according to defined clinical indicators and fills out questionnaires.

The recruitment process is continued until the necessary number of eligible participants are included in the intervention and control groups within the following two target populations: (1) obese citizens at risk of developing a chronic disease and (2) obese patients with type 2 diabetes.

**Inclusion and exclusion criteria.**

**Inclusion criteria**

- BMI ≥30 but ≤45 kg/m^2^

- Age 18-70 years

**Exclusion criteria**

- No informed consent

- No completion of the initial questionnaire

- No internet access in own home through a computer or smartphone

- Pregnancy or active attempts to get pregnant

- Serious or life-threatening disease

### Randomization of Participants

**Figure 1:**
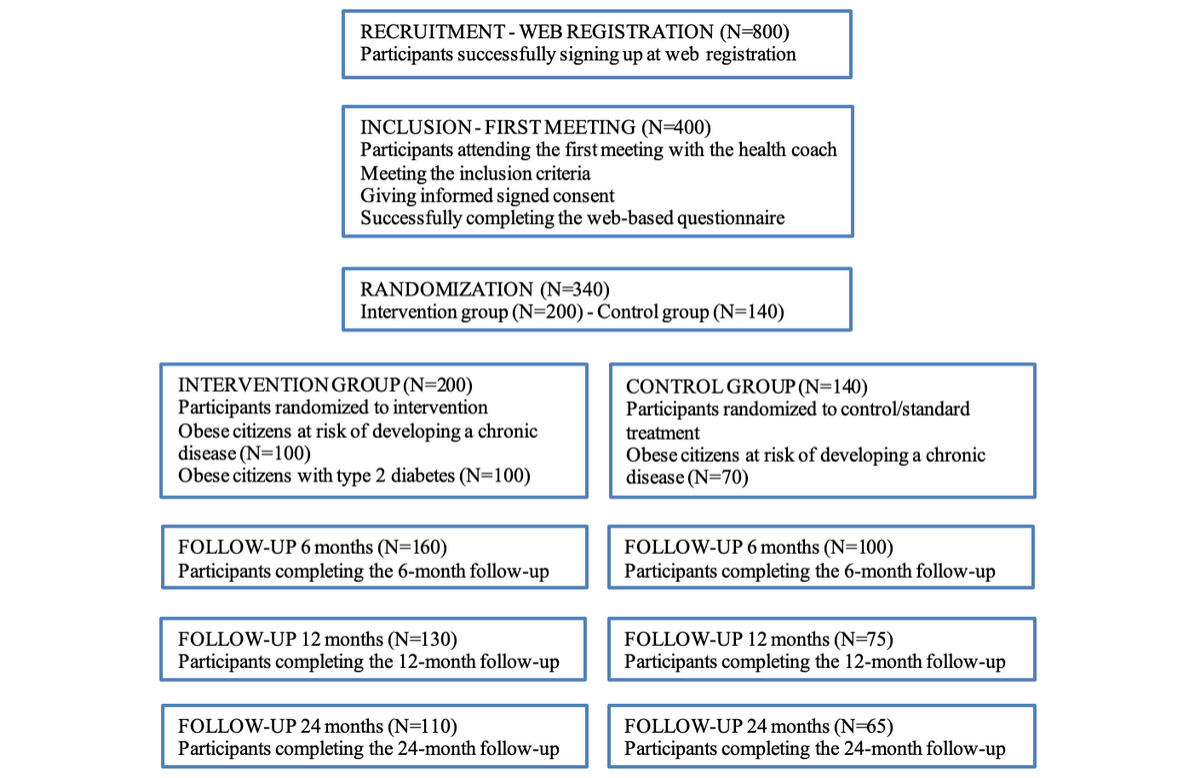
Illustration of participant flow.

After all participants successfully complete a web-based questionnaire and undergo baseline measurements, they are randomized via an automated computer algorithm. This procedure ensures that drop-out characteristics can be recorded. Participants are randomized in a 60:40 manner, where 60% of the recruited participants are randomized to the intervention group and the remaining 40% are included in the control group*.* Randomization is controlled to ensure that 50% of participants in the intervention group and control group will be obese citizens, who have not been previously diagnosed with type 2 diabetes, and the other 50% of participants in the intervention group and control group will be patients diagnosed with type 2 diabetes (BMI ≥30 but ≤45 kg/m^2^) (Figure 1).

### Ethics

The intervention is not expected to cause any side effects or discomfort. The only recognized risk is in relation to eating disorders (pre-existing or developing during the study), and the dieticians and nurses involved in the study will specifically check for the indications. If an eating disorder is detected, the participant in question will be excluded from the study. The intervention is offered in addition to standard municipal care service, and participants can withdraw from the program at any time. The Regional Ethical Committee has approved the study according to Danish law (no. 18803). Participant data will be handled and stored in accordance with rules approved by the Danish Data Protection Agency. Permission to handle individual participant data from the national registries will be obtained from the Danish Data Protection Agency. All data will be analyzed in anonymized forms.

### Intervention

Participants in the intervention group receive login information for LIVA 2.0 at the first personal face-to-face meeting (either physical or digital), after which the health coach introduces the LIVA 2.0 app. After the first personal meeting, the same health coach will be coaching the participant throughout the period. When a health coach is on vacation or is sick for a short period, coaching is postponed. If the health coach is sick for a long period, a personal meeting with a new health coach is arranged to secure a personal relationship, after which the new health coach will be coaching the participant for the rest of the period. Based on our experiences from earlier studies, a half-time health coach can manage between 200 and 250 individuals. The participant and health coach together agree on goals for diet, physical exercise, sleep, and all other life areas of relevance to the participant. All goals are participant driven on the basis of the initial motivational interview with the health coach. The health coach is required to identify what health initiatives will benefit the participant the most based on the participant’s own wishes and to find out what is possible for the participant taking into account the participant’s personal barriers and facilitators. Using the app, the participant provides a daily record and also enters comments, concerns, and questions for the health coach, who can see the entire profile of the participant. The health coach provides individual asynchronous online consultation according to the participant’s needs and based on the participant’s registrations. The health coach encourages and praises goal attainment and endeavors to keep the participant motivated. Additionally, the health coach provides advice related to setting goals based on the SMART model (specific, measurable, attainable, relevant, and timely) [29] according to a predefined guideline structure (Table 1) [27,29].

**Table 1:** Template of the Intervention Description and Replication checklist [30] for the eHealth lifestyle coaching tool (LIVA 2.0).

Item	Description
eHealth coaching sessions	Prior to the intervention, the health coaches receive training in setting SMART (specific, measurable, agreed upon, realistic, and time-based) goals [29] with the participants using the eHealth solution LIVA 2.0 and in setting up action and coping plans that address barrier identification and problem solving. Participants in the intervention group have one or two personal meetings (face-to-face or digital) with their health coach, followed by asynchronous web-based consultations based on dialog by means of text or video. The consultations address the participant’s registrations, goal setting, and questions regarding diet, exercise, and lifestyle plans, taking into consideration chronic diseases. The LIVA 2.0 app is set up with short explanations about different functions, and notifications and reminders to the participants to register and give feedback about the health coaching. The sessions provide the participant with information in relation to their status, specific focus on goals, and recommendations on how to improve their behaviors. Include BCTs^a^ from CALO-RE^b^ taxonomy [31] (hereafter referred to as BCTs) as follows: provide information on the consequences of the behavior in *general* and *to the* *individual*, goal setting, behavior and outcome, action planning, and barrier identification/problem solving; set graded tasks; prompt review of behavioral goals; prompt review of outcome goals; prompt rewards contingent on effort or progress toward behavior; prompt generalization of a target behavior; and provide feedback on performance (Multimedia Appendix 1).
Goals and inputs	Goals and inputs are always driven by the participant and are available to the participant, who can choose the focus area, set specific goals, and keep a record of specified behaviors by reporting them on a daily, weekly, or monthly basis. This allows the user and the health coach to follow progress or setbacks as the numbers and registrations are visualized using graphs and curves. All coaching by the health coach follows national guidelines from the Danish National Board of Health.
Dietary goals and plans	Dietary goals and plans can be set at many different levels (eg, from simple changes aiming at changing one meal a day to more complex changes aiming at a completely new diet for the remedying of digestion problems).
Physical activity goals and plans	Physical activity goals and plans involve goal setting and recording of the type of physical activity and time for executing the given physical activity. The participant receives advice and/or a video on activities in a variety of contexts to foster physical activity as a more integrated part of life (BCT: provide instruction on how to perform the behavior, prompt generalization of a target behavior, and provide relapse prevention/coping planning).
Life goals	Goals on a healthy joyful life as the participant sees it (eg, daily life with less stress, stronger social bonds with friends and family, coping skills for diseases, etc).
Weight	Set the current weight and goal for a lower or higher weight and register new measurements on a daily, weekly, or monthly basis.
Steps	When downloading the LIVA 2.0 app, the participant can accept direct import of the information on steps recorded on a smartphone, and tailored messages on progress toward a set goal appear simultaneously (BCT: teach-to-use prompts/cues). Step count monitoring is encouraged but not required to enter the LIVA study. Some participants will have other ways of registering their physical activity level.
Pain, sleep, and mood	Give daily feedback on pain, sleep, and mood, which can affect the ability to perform a given behavior (BCT: relapse prevention/coping planning).
Smoking	Set goals to bring down the number of cigarettes smoked on a daily basis, leading to cessation.
Blood glucose and blood pressure	Keep a record of specified measures expected to be influenced by the different behavior changes addressed. In LIVA, this includes blood glucose and blood pressure measurements (BCT: prompt self-monitoring of behavioral outcome and provide information on consequences of the behavior in *general* and *for the* *individual*).
Forum	Online forum where the participant can exchange knowledge, gain social support, and build new relationships, and the health coach can provide advice to the participant (BCT: plan social support/change).
Coaching providers	Health coaches with basic training as nurses, physiotherapists, dieticians, or occupational therapists. In Denmark, all four education types consist of 420 European Credit Transfer System (ECTS) points (3.5 years of full-time education). In addition to their education as health care professionals, they all undergo special training in using digital health coaching and practice digital health coaching for at least 2 years.
Coaching approach	Individually delivered via the app or web-based delivery.
Coaching location	Initial personal meetings in municipality health centers, general practice medical centers, or the research unit for general practice at the University of Southern Denmark or over the internet, and then, solely web-based delivery.
Coaching time and quantity	The initial consultation with a health coach is estimated to last for approximately 45-60 minutes. The subsequent asynchronous eHealth coaching sessions are carried out once a week in the first 6 months and then every month for the last 6 months for maintenance. Thereafter, the participant could receive two eHealth coaching sessions and use LIVA 2.0 as a personal BCT tool (BCT: use of follow-up prompts).
Tailoring	Every participant receives personalized eHealth coaching sessions from their designated health coach. The provided feedback is based on the participant’s inputs on LIVA 2.0.

^a^BCT: behavior change technique.

^b^CALO-RE: Coventry, Aberdeen and LOndon-REfined taxonomy [31].

Asynchronous online consultations are held weekly during the first 6 months. Thereafter, online consultations are held monthly over a period of 6 months. After 12 months, the participants enter a retention period for 12 months, where the health coach will follow the participants’ registrations and may provide up to a maximum of four coaching sessions during that year. We expect that approximately 20% of participants will not be ready for retention after 1 year and these participants will be offered yet another year of intervention. This evaluation is performed by the health coach. The health coach endeavors to maintain participation through phone calls, text messages, and, if necessary, face-to-face meetings to prevent drop out*.* After 24 months of follow-up, participants are followed through national registers for long-term follow-up on a number of predefined endpoints. Participants in the intervention group receive the standard municipal preventive care service, such as diabetes education, to the extent that municipalities normally provide these care services throughout the observation period.

### Conventional Care Service (Control Group)

Participants randomized to the control group are offered to receive the standard municipal secondary or tertiary preventive care service. A recent study aimed at examining Danish municipal weight loss care services and identifying and describing their content and structure found 234 different municipal weight loss care services. Although they were different, they most frequently contained information about diet and physical activity. They also sometimes included information about how to develop healthy habits, and a few of the care services even included the promotion of well-being and social participation [32]. The standard municipal care service within the control group is therefore not the same, but none of the care services resembled the LIVA 2.0 program. At follow-up measurements, the control group participants were asked to describe if they had participated in any interventions since baseline, and if so, describe the content of these interventions. This enabled a qualitative assessment and summary of the standard care services used in the control group.

### Outcome Measures

Measurements are conducted by the health coach at baseline and at the 6-, 12-, and 24-month follow-ups. Data from national registers are collected before baseline and up to 3 years after the end of the intervention. Measurements are collected in a facility provided by the local municipality, the local general practitioner, or the clinical research center. Participants are scheduled for appointments by phone and with confirmation through e-mail. The facilities consist of a private consultation room with measurement equipment calibrated for use at relevant timelines and a waiting room area. Multimedia Appendix 2 describes all the included variables, definitions, categories, and sources for both assessments and explaining variables. No data from the participant records are obtained directly, and they are obtained only indirectly through registrations in national registers.

#### Clinical Assessments

Weight and HbA_1c_ are measured by the health coach using standard and validated measurement equipment (Tanita BC 420 S MA). Height, waist circumference, and hip circumference are measured, and BMI is calculated from the measured weight and height.

#### Lifestyle Assessment

QOL is measured through a validated questionnaire instrument (12-item Short Form Survey [SF-12]) [33].

#### Health Economic and Long-Term Assessments

Health care service usage, pharmaceutical consumption, and consumption of municipal care services, as well as morbidity status and mortality are measured through register data. Data from the Danish National Participant Register [34], the Danish National Prescription Registry [35], the Danish National Health Service Register [36], the Danish Civil Registration System [37], and relevant municipal statements and registers will be linked. Data linkage between registers is possible using the unique Danish Personal Identiﬁcation Number, which is assigned to each Danish citizen and applied throughout the public and private sectors [37]. Productivity loss is evaluated through a questionnaire (Multimedia Appendix 2) [38].

#### Explaining Variables

Each participant’s sociodemographic characteristics are obtained at the baseline face-to-face meeting and registered by the health coach. The explaining variables are used in the descriptive analyses of associations between the participants’ characteristics and the success of using LIVA 2.0.

### Health Economic Design

Within the scope of a cost-effectiveness analysis, expenditures related to the intervention, including acquisition, deployment, and operational costs, are compared with outcomes of the intervention in relation to the defined assessments. The cost effectiveness of the intervention is primarily assessed from the perspectives of the municipalities. The total cost of the intervention paid by a Danish municipality consists of investment costs as well as operating costs. Investment costs include expenditure for the training of health professionals as well as basic preparation cost. Operating costs cover the annual license fees for the individuals participating in the intervention as well as the individuals in the 1-year retention period. It is assumed that after 1 year in the intervention, participants will move to the retention period, where they will remain for 1 year; however, every year, 20% of the initial population will ultimately leave the intervention. Additionally, the operating costs include the salaries of the health professionals. The costs are compared to the effect of the intervention to evaluate cost effectiveness both in relation to the clinical effect and QOL [39].

### Budget Impact Analysis

A municipal budget impact analysis is performed, subtracting possible savings for the municipality in relation to health care costs, nursing costs, and lost productivity from the investment costs [40]. As part of the budget impact analysis, the annual rate of return of municipality investments is estimated. Follow-up data covering 1 year before baseline until 3 years after are collected from national registers (Multimedia Appendix 2). To investigate the possible impact of the intervention on health care service usage by participants, pharmaceutical consumption and municipal service usage as annual consumption are compared to a reference year 1 year before the intervention (baseline minus 1 year).

As part of the analysis, the following two scenarios are examined: (1) A_0_, baseline scenario, where no intervention has been introduced. The health status of the examined population is expected to decrease, whereas the municipality costs are expected to increase; (2) A_1_, alternative scenario, where the eHealth lifestyle coaching intervention has been introduced. The health status of the examined population is expected to increase owing to the intervention, whereas municipal costs are expected to decrease.

### Long-Term Follow-Up

Since the target group involves participants with chronic diseases or having a risk of chronic diseases, the effect structure of a lifestyle change will first be visible over several years. These data will not be available within the current study design, and hence, the observed effects within the given time frame will be extrapolated over time. We will develop hypotheses for the effect structure according to the literature to be able to model the long-term (5 years) effect of the intervention [41,42].

### Analysis Strategy

All data are analyzed according to the intention-to-treat principle [43]. Analyses are mainly carried out according to the two target groups. However, stratification according to participant characteristics and user experiences is applied to investigate associations between success and the different characteristics. Ordinary least square regression and difference between groups over time are used to explore significant associations. Statistical significance is inferred at a two-tailed *P* value <.05. Robust standard errors are calculated. Data are analyzed by experts in biostatistics. After the study, all data will be made accessible on request in anonymized form to allow full peer scrutiny and facilitate secondary research.

### Sample Size Considerations

The primary objective of this study is the measurement of changes in body weight and waist circumference. Based on a recent study by Haste et al, which evaluated a web-based weight loss intervention among men with diabetes, we expect a weight loss of at least 4.5 kg at 12 months in the intervention group as compared with 2.5 kg in the control group [8] (Textbox 2).

**Sample size calculation.**

A power calculation based on standard deviations observed in the study [8] shows that the detection of a difference in weight loss of 2.0 kg with a power of 0.95% requires 55 participants in the intervention group and 32 in the control group. To allow for drop out according to the experienced attrition rates in the same study [8] (39% of participants in the intervention group and 57% in the control group are expected to drop out at 12 months), we will recruit 100 participants in the intervention group and 70 in the control group (Figure 1). To be able to stratify analyses according to obese participants at risk of developing chronic diseases and obese participants with diabetes, we will recruit 100 obese participants at risk of developing chronic diseases and 100 obese participants with diabetes. Therefore, in total, we will consider 200 participants in the intervention group and 140 in the control group.

### Prevention of Drop Out and Loss to Follow-Up

Based on prior experience, approximately 15% of participants will drop out during the first 2 weeks owing to technological challenges, etc. Likewise, after 3 months, approximately 20% of participants will lose motivation and be less active on the platform [8]. The coach will endeavor to maintain participation through phone calls, text messages, and, if necessary, face-to-face meetings. In the case of exclusion before the end of the trial (eg, due to pregnancy), the participant will be asked to complete a final questionnaire and have objective parameters measured in order to provide data for the intention-to-treat analysis.

## Results

### Progress

From March 2018 to March 2019, 799 potential participants were evaluated for participation. A total of 340 met the inclusion criteria, consented to participate, filled out the web-based questionnaire, and were randomized into study groups. Among participants with type 2 diabetes, 100 (49 female participants) were randomized to the intervention group and 70 (32 female participants) were randomized to the standard care group. Among overweight participants, 100 (81 female participants) were randomized to the intervention group and 70 (52 female participants) were randomized to the standard care group.

### Findings

The hypothesis is that the intervention group will demonstrate decreased body weight and a much higher percentage of patients with normalization of their HbA_1c_ levels as compared with the control group. A relevant percentage of participants in the intervention group are expected to decrease their use of glucose-lowering medications and improve their QoL much more as compared with the control group. Operational costs are expected to be lower than standard care and the intervention is expected to be cost-effective for the intervention group.

### Timeline

The first baseline assessments were carried out in March 2018. The trial has now reached the 12-month follow-up period for all included participants, and results are expected by the middle of 2020. The 2-year follow-up will be carried out between March 2020 and April 2021.

## Discussion

This is the first time an app and web-based eHealth lifestyle coaching program implemented in Danish municipalities will be clinically and economically evaluated in a strong scientific setup. The study is expected to show that human support through a digital lifestyle intervention program leads to relevant weight loss as compared with a control group receiving standard care, and more importantly, the study is expected to show that digital lifestyle support results in more than twice as many patients with type 2 diabetes reaching a relevant weight loss of 3% to 5% or more [8]. These results are expected to be clinically relevant for patients with type 2 diabetes when weight loss is sustained for more than 2 years [44]. Moreover, these findings will support that the remission rates of patients with type 2 diabetes can be improved by the use of digital lifestyle coaching at a level comparable to other more intensive resource-heavy strategies [5,7]. If the LIVA 2.0 eHealth lifestyle coaching program is proven to be effective, there is a great potential for decreasing obesity rates and rehabilitating patients with type 2 diabetes and other related chronic diseases cost-effectively though human digital lifestyle coaching.

## Appendix

Multimedia Appendix 1 YouTube video showing an example of the LIVA app used in real life.

Multimedia Appendix 2 Included variables, definitions, categories, and source.

## Abbreviations

BCT: behavior change technique
DKK: Danish Krones
HbA_1c_: hemoglobin A_1c_
LIVA: long-term Lifestyle change InterVention and eHealth Application
QOL: quality of life
RCT: randomized controlled trial
SF-12: 12-item Short Form Survey
SMART model: specific, measurable, attainable, relevant, timely
