# Supplementary material for: Evaluation of the Clinical and Economic Effects of a Primary Care Anchored, Collaborative, Electronic Health Lifestyle Coaching Program in Denmark: Protocol for a Two-Year Randomized Controlled Trial
Source: JMIR Res Protoc. 2020 Jun 25;9(6):e19172. doi: 10.2196/19172 (PMC7380992; doi:10.2196/19172)
Supplement: Multimedia Appendix 1 [file resprot_v9i6e19172_app1.docx]

**NEW SUPPLEMENTARY FILE:**

**youtube video showing an example of the LIVA app used in real life, as requested by one of the reviewers**

<https://www.youtube.com/watch?v=-dlpN1znH7U&feature=emb_logo>
