# Supplementary material for: Evaluation of the Clinical and Economic Effects of a Primary Care Anchored, Collaborative, Electronic Health Lifestyle Coaching Program in Denmark: Protocol for a Two-Year Randomized Controlled Trial
Source: JMIR Res Protoc. 2020 Jun 25;9(6):e19172. doi: 10.2196/19172 (PMC7380992; doi:10.2196/19172)
Supplement: Multimedia Appendix 2 [file resprot_v9i6e19172_app2.docx]

**Appendix 2.** Included variables, definitions, categories, and source.

| **Characteristics** | | **Definitions / measurements** | **Categories** | **Source** |
| --- | --- | --- | --- | --- |
|  | |  |  |  |
| **Demographics and socioeconomics** | |  |  |  |
|  | Sex | Gender | (1) Male  (2) Female | Baseline face-to-face meeting with the health coach |
|  | Age | Age in mid-year | Continuous | Baseline face-to-face meeting with the health coach |
|  | Highest educational level attained | Highest educational level attained at the date of data extraction, based on the main groups in the Danish educational nomenclature with 13 educational groups based on years of education | Variable with three categories  (1) Primary education (<11 years)  (2) Middle high education (11-15 years)  (3) Higher education  (16+ years) | Baseline face-to-face meeting with the health coach |
|  | Income level | Annual gross income in 2018 and employee benefits | Continuous variable or categorical variable with three categories  (1) DKK^a^ 149,999 or less  (2) DKK 150,000-349,999  (3) DKK 350,000 or more | Baseline face-to-face meeting with the health coach |
|  | Civil status | Marital status | (1) Married or in civil partnership  (2) Unmarried  (3) Widow or longest living partner  (4) Divorced or cancelled partnership | Baseline face-to-face meeting with the health coach |
|  | Occupational status | Affiliation to the labor market | (1) Affiliated to the labor market (employed or self-employed)  (2) Unemployed (maternal leave or job seeker allowance)  (3) Unemployed (unemployment benefit)  (4) Education  (5) Early retirement  (6) Retired  (7) Child | Baseline face-to-face meeting with the health coach |
|  | Municipality | Municipality of residence |  | Baseline face-to-face meeting with the health coach |
| **Clinical indicators** | |  |  |  |
|  | HbA1c^b^ | Finger blood sample analyzed for glycated hemoglobin. Indicates average blood glucose levels over the past two months. Blood samples are analyzed using a HemoCue HbA1c 501 machine | Continuous variable measured in mmol/mol | Baseline and follow-up meetings with the health coach |
|  | BMI | Weight (with clothes but without shoes plus 1 kg) divided by squared height (without shoes) both measured in accordance with the EHES guideline (<http://www.ehes.info/manuals.htm>) | Continuous variable measured in kg/m^2^ | Baseline and follow-up meetings with the health coach |
|  | Waist and hip circumference | Measurement with tape measure around the waist (between lower rib and pelvic curvature) and hip (one hand above the inguinal medial line) in accordance with the EHES guideline (http://www.ehes.info/manuals.htm) | Continuous variable measured in cm | Baseline and follow-up meetings with the health coach |
| **Lifestyle indicators** | |  |  |  |
|  | Quality of life | SF-12^c^ score | SF-12 score | Questionnaire at baseline and follow-up meetings with the health coach |
| **Health economic and long-term indicators** | |  |  |  |
|  | Usage of secondary care services | Inpatient and outpatient services delivered in Danish hospitals registered in the National Participant Register divided into the following components:  (1) Inpatient services  (2) Inpatient services for stays longer than the average participant in this DRG^d^ group  (3) Inpatient services for rehabilitation  (4) Outpatient services  (5) Outpatient services for stays longer than the average participant in this DAGS^e^ group  (6) Outpatient services for rehabilitation  DRG system and DAGS tariffs. The DRG tariff system is developed for administrative purposes and is based on estimated average costs across hospitals for specific diagnostic groups. Excludes interest and depreciation of buildings and equipment, while other overhead costs are included. | Continuous variables measured in DKK | DRG/DAGS grouped National Participant Register annually up to 3 years after the study |
|  | Usage of primary care services | Primary care services delivered by general practitioners and privately practicing specialists, such as dentists, physiotherapists, chiropractors, and chiropodists, who are registered in the National Health Service Register, which are divided into the following components:  (1) Services in general practice  (2) Services for privately practicing specialists  Reimbursement fees between the National Health Insurance scheme and private practicing physicians are used as cost units. General practitioners are compensated by administrative regions through a combination of per capita fee (approximately 30% of the total) and fee for service (approximately 70%) [38]. To reflect this payment scheme in the unit cost, 43.8% of the fee for services in general practice is added. Overhead costs covered by capitation fee are hence not distributed across the numbers of visits, as would have been most appropriate, but by resource burden. | Continuous variables measured in DKK | The Health Insurance Register annually up to 3 years after the study |
|  | Usage of prescribed pharmaceuticals | Prescribed pharmaceuticals dispensed by Danish pharmacies and registered in the Danish National Prescription Register (pharmaceuticals consumed in hospitals are included in DRG tariffs. Over-the-counter drugs are not included in the statements).  Total sale price includes participant out-of-pocket payments since costs of prescribed pharmaceuticals are shared between the participants and the primary health care sector by a copayment scheme where participants are reimbursed according to the need. These costs are aggregated since total costs are measured regardless of who pays, and 20% VAT is subtracted | Continuous variables measured in DKK | Prescription register annually up to 3 years after the study |
|  | Municipality services: Nursing services | Has the participant received nursing services through the municipality? | Continuous variable measured in minutes | Registers in the municipalities annually up to 3 years after the study |
|  | Municipality services: Rehabilitation | Has the participant received rehabilitation services through the municipality? | Continuous variable measured in minutes | Registers in the municipalities annually up to 3 years after the study |
|  | Presenteeism | How often in the past 2 weeks have you felt that you were not able to perform 100% at your work? | Continuous variable | Questionnaire at baseline and follow-up meetings with the health coach |
|  | Days of absence owing to illness | How many days in the past month have you been out of work due to illness? | Continuous variable | Questionnaire at baseline and follow-up meetings with the health coach |

^a^1 DKK=0.134 Euro (€) = 0,149 US$.

^b^HbA1c: hemoglobin A1c.

^c^SF-12: 12-item Short Form Survey.

^d^DRG: Diagnosis Related Grouping.

^e^DAGS: Danish Ambulant Grouping System.
